# Lactate Kinetics Reflect Organ Dysfunction and Are Associated with Adverse Outcomes in Intensive Care Unit Patients with COVID-19 Pneumonia: Preliminary Results from a GREEK Single-Centre Study

**DOI:** 10.3390/metabo10100386

**Published:** 2020-09-28

**Authors:** Alice G. Vassiliou, Edison Jahaj, Ioannis Ilias, Vassiliki Markaki, Sotirios Malachias, Charikleia Vrettou, Eleni Ischaki, Zafeiria Mastora, Evangelia Douka, Chrysi Keskinidou, Stamatios Tsipilis, Dimitra A. Vassiliadi, Anastasia Kotanidou, Ioanna Dimopoulou

**Affiliations:** First Department of Critical Care Medicine & Pulmonary Services, Medical School of National & Kapodistrian University of Athens, Evangelismos Hospital, 10676 Athens, Greece; alvass75@gmail.com (A.G.V.); edison.jahaj@gmail.com (E.J.); ineotis@endo.gr (I.I.); vas.markaki@gmail.com (V.M.); sotmalachias@gmail.com (S.M.); Kliovrettou@med.uoa.gr (C.V.); eleniischaki@gmail.com (E.I.); zafimast@gmail.com (Z.M.); edouka@evaggelismos-Hosp.gr (E.D.); chrysakes29@gmail.com (C.K.); stamostsipil@gmail.com (S.T.); dimitra.vas@gmail.com (D.A.V.); akotanid@med.uoa.gr (A.K.)

**Keywords:** COVID-19, acute respiratory distress syndrome, lactate, SOFA, mortality

## Abstract

Coronavirus disease-19 (COVID-19) continues to be a health threat worldwide. Increased blood lactate is common in intensive care unit (ICU) patients; however, its association with outcomes in ICU COVID-19 patients remains currently unexplored. In this retrospective, observational study we assessed whether lactate is associated with outcomes in COVID-19 patients. Blood lactate was measured on ICU admission and thereafter daily up to day 14 in 45 patients with confirmed COVID-19 pneumonia. Acute physiology and chronic health evaluation (APACHE II) was calculated on ICU admission, and sequential organ failure assessment (SOFA) score was assessed on admission and every second day. The cohort was divided into survivors and non-survivors based on 28-day ICU mortality (24.4%). Cox regression analysis revealed that maximum lactate on admission was independently related to 28-day ICU mortality with time in the presence of APACHE II (RR = 2.45, *p* = 0.008). Lactate’s area under the curve for detecting 28-day ICU mortality was 0.77 (*p* = 0.008). Mixed model analysis showed that mean daily lactate levels were higher in non-survivors (*p* < 0.0001); the model applied on SOFA scores showed a similar time pattern. Thus, initial blood lactate was an independent outcome predictor in COVID-19 ICU patients. The time course of lactate mirrors organ dysfunction and is associated with poor clinical outcomes.

## 1. Introduction

An outbreak of an unknown infectious pneumonia occurred recently in Wuhan, China. The pathogen of the disease was quickly identified as a novel coronavirus (SARS-CoV-2, severe acute respiratory syndrome coronavirus 2), and the disease was named coronavirus disease-19 (COVID-19) [1]. The disease rapidly spread throughout the world and COVID-19 is now an emerging health threat. The pathogenesis of COVID-19 is not completely understood; however, it seems that excessive immune responses, together with lytic effects of the virus on host cells, play a crucial role [2]. The clinical spectrum of COVID-19 infection ranges from mild upper respiratory tract illness to severe pneumonia with respiratory failure requiring treatment in an intensive care unit (ICU). In China, between 24 December 2019 and 26 January 2020, of the 710 patients, 52 patients (7%) were admitted to the ICU [3]. In Italy (Lombardi, between 20 February and 18 March 2020), of the 17,713 people who were positive for the virus, 1591 patients (9%) required ICU support [4]. In the New York City area, a study which included all sequentially hospitalized patients between 1 March 2020, and 4 April 2020, showed that out of 5279 patients, 647 patients (12%) were treated in the ICU [5], and in the UK, out of 18,133 patients, 3001 (17%) [6], were admitted to the ICU between 6 February and 19 April 2020.

Increased blood lactate concentration (hyperlactatemia) is common in critically ill patients [7,8]. Arterial blood lactate levels are routinely measured in the ICU to estimate disease severity, predict morbidity and mortality, indicate specific treatments and monitor the adequacy and timing of interventions [7]. Vincent and colleagues first introduced the concept of serial lactate measurements during shock. Evaluation of the time course of blood lactate concentrations may yield more information than a single blood lactate measurement [9]. Several investigators continued to study the time variable in lactate kinetics [10,11,12]. In patients with septic shock, the lactime—the time during which lactate levels remain >2 mmol/L—is the best outcome predictor [11]. Similarly, in trauma patients, lactate normalization within 24 h is associated with 100% survival [10]. The third international consensus definition for sepsis and septic shock recently revised the definition of septic shock. Serum lactate concentration >2 mmol/L was added as a key component in the definition [13]. Despite these, the association between lactate concentrations and outcomes remains currently unexplored in ICU patients with COVID-19 pneumonia.

The aim of the present study was to ascertain if arterial blood lactate on admission in the ICU predicts 28-day mortality in patients with COVID-19 pneumonia, and to identify a cut-off value that best correlates with mortality. Furthermore, we wished to evaluate whether the time course of blood lactate concentrations is associated with outcome. For this purpose, lactate was measured on admission in the ICU and daily up to day 14. The present retrospective, observational study was conducted at the Evangelismos academic General Hospital, Athens, Greece, the biggest of the five referral centers for COVID-19 in Athens.

## 2. Results

During the study period, 122 patients with confirmed COVID-19 infection were admitted to the hospital. Of these, 45 patients (37%) required treatment in the ICU. The cohort consisted of 33 males and 12 females, with a mean age of 64 ± 10 years. Sixty-seven percent suffered from comorbidities, with the most common being arterial hypertension (*n* = 16). All 45 patients had an abnormal chest radiograph showing bilateral infiltrates on chest X-Rays. The most common symptoms prior to ICU admission were fever, cough and dyspnea. All patients were admitted for hypoxemic respiratory failure, and 7/45 patients (16%) had also septic shock, receiving norepinephrine at a dose ranging from 0.2 to 1.3 μg/kg/min. Forty of the 45 patients (89%) were mechanically ventilated, and five patients (11%) received high-flow oxygen therapy (>15 L/min). Forty-three patients (96%) had acute respiratory distress syndrome (ARDS), seven patients had severe ARDS (PaO_2_/FiO_2_ < 100 mmHg), 25 patients had moderate ARDS (PaO_2_/FiO_2_ < 200 mmHg), and 11 patients had mild ARDS (PaO_2_/FiO_2_ between 200–300 mmHg). In two patients PaO_2_/FiO_2_ was 348 and 352 mmHg, respectively. Hyperglycemia (glucose above 180 mg/dL) was common in the cohort [14]. The median admission acute physiology and chronic health evaluation (APACHE II) and sequential organ failure assessment (SOFA) scores were 17 (IQR: 14–19) and 9 (IQR: 8–10), respectively. COVID-19-targeted treatment included azithromycin, chloroquine and lopinavir/ritonavir (*n* = 20); azithromycin and chloroquine (*n* = 15); lopinavir/ritonavir and chloroquine (*n* = 5); or chloroquine alone (*n* = 4). One patient received plasma from a recovered patient. In 37/45 patients (82%) broad-spectrum antibiotics were concomitantly administered.

The cohort was divided into survivors and non-survivors, based on 28-day ICU mortality. Eleven of the 45 patients died, yielding a mortality rate of 24.4%. Demographics, illness severity scores and important outcomes of the two patient groups are summarized in supplemental Table S1. As seen in Table 1, on ICU admission, APACHE II, SOFA score and lactate levels were significantly higher in non-survivors compared to survivors. Other variables that differed between the two groups were respiratory rate, temperature, urea, creatinine, high-sensitive troponin T, hemoglobin and the hematocrit. The results of univariate Cox regression analysis are shown in Table 2. The multivariate model identified maximum admission lactate as an independent outcome predictor for 28-day ICU mortality with time (RR = 2.448, 95% CI = 1.267–4.730, *p* = 0.008), in the presence of APACHE II (RR = 1.178, 95% CI = 0.961–1.443, *p* = 0.11).

**Figure 1:**
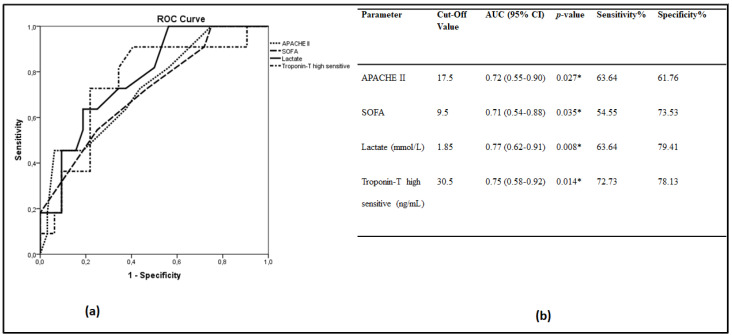
Lactate levels and 28-day mortality. (**a**,**b**) Receiver operating characteristic (ROC) curve analysis. ROC curves were generated to determine the prognostic accuracy of APACHE II, SOFA, high-sensitive troponin T and lactate, measured on ICU admission. (**a**) ROC curves; APACHE II, dotted line; SOFA, dashed line; high-sensitive troponin T, dash-dotted line; lactate, solid line; (**b**) The corresponding areas under the curve (AUC), 95% confidence intervals (CI) and the optimal cut-off values with the greatest combined sensitivity and specificity are given. * *p*-value < 0.05. Lactate measurements of days 1 and 2 were combined because admission to the ICU occurred mostly in late afternoon. APACHE = acute physiology and chronic health evaluation; SOFA = sequential organ failure assessment.

Receiver operating characteristic (ROC) curves were generated to determine the prognostic accuracy of blood lactate, APACHE II, SOFA score and troponin T in our cohort (Figure 1a). The area under the ROC curve (AUC) and 95% CI for detecting the main outcome, i.e., 28-day ICU mortality, were estimated (Figure 1b). Lactate at 1.85 mmol/L showed a sensitivity of 64% and a specificity of 79.4%. The positive likelihood ratio (PLR) was 3.09, whereas the negative likelihood ratio (NLR) was calculated at 0.46. Pair-wise comparisons of the ROC curves revealed that lactate, APACHE II, SOFA and troponin T were equal in predicting 28-day ICU mortality.

**Figure 2:**
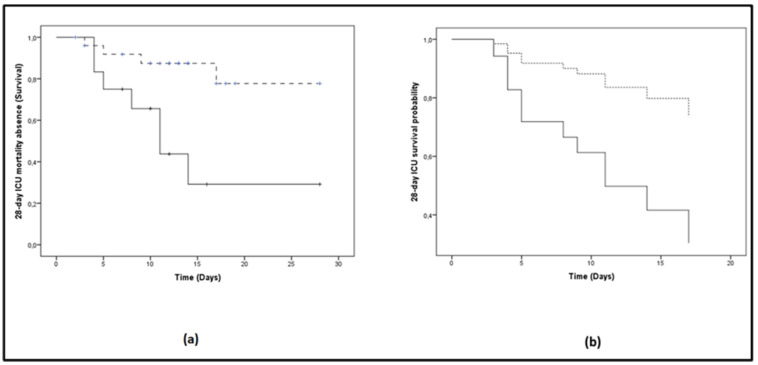
Lactate levels on ICU admission and 28-day mortality probability. The patient cohort was dichotomized above and below the best lactate prognostic discriminating value. Solid lines: ≥1.85 mmol/L; dashed lines: <1.85 mmol/L. (**a**) The Kaplan–Meier method was used for 28-day ICU mortality absence probability estimation and the log-rank test for comparison of the two groups. The respective mean time to 28-day ICU mortality was 14.4 days (95% CI: 8.5–20.3) for the high lactate group, whereas for the low lactate group it was 24.1 days (95% CI: 20.7–27.6) (log-rank test, *p* = 0.005); (**b**) The corresponding Cox proportional hazards regression model also showed a significant difference in survival with lactate over (solid line) or under (dashed line) the cut-off of 1.85 mmol/L (RR: 0.257 (0.077–0.857), *p* = 0.027). Lactate measurements of days 1 and 2 were combined because admission to the ICU occurred mostly in late afternoon.

When the whole cohort was dichotomized above (≥1.85 mmol/L) and below the best lactate prognostic discriminating value (<1.85 mmol/L), as determined from the ROC curve, the probability of 28-day ICU mortality was significantly elevated in the high lactate group (log-rank test, *p* = 0.005; Figure 2a). The respective mean time to ICU mortality was 14.4 days (95% CI: 8.5–20.3) for the high lactate group and 24.1 days (95% CI: 20.7–27.6) for the low lactate group. The corresponding Cox proportional hazards regression model also showed a significant difference in survival with lactate over or under the cut-off of 1.85 mmol/L (RR: 0.257 (0.077–0.857), *p* = 0.027; Figure 2b).

**Figure 3:**
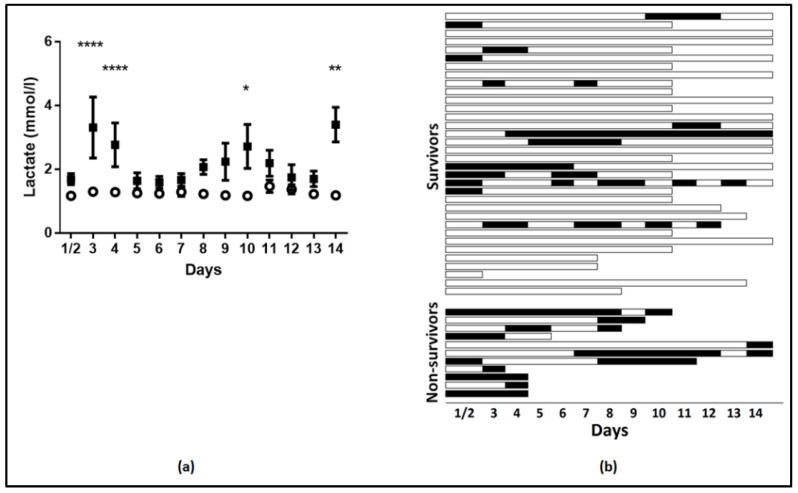
Mean lactate levels per day in survivors and non-survivors. (**a**) Mean daily lactate levels and 28-day ICU mortality. Analysis was performed using mixed model fixed effects analysis for the mean daily levels of lactate over 14 days and 28-day ICU mortality as the grouping factor. Open circle, survivors; closed square, non-survivors. * *p* < 0.05, ** *p* < 0.01, **** *p* < 0.0001; (**b**) Hyperlactatemia (>2 mmol/L) over 14 days in the ICU; hyperlactatemia is depicted with black boxes. Every horizontal line represents one patient. Lactate measurements of days 1 and 2 were combined because admission to the ICU occurred mostly in late afternoon.

**Figure 4:**
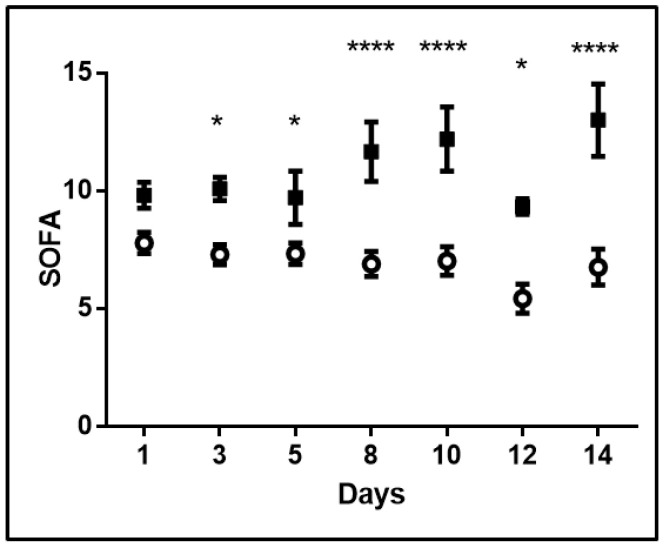
SOFA scores in survivors and non-survivors. SOFA scores and 28-day ICU mortality. Analysis was performed using mixed model fixed effect analysis for SOFA scores measured on admission, and days 3, 5, 8, 10, 12, and 14, using 28-day ICU mortality as the grouping factor. Open circle, survivors; closed square, non-survivors. * *p* < 0.05, **** *p* < 0.0001.

**Figure 5:**
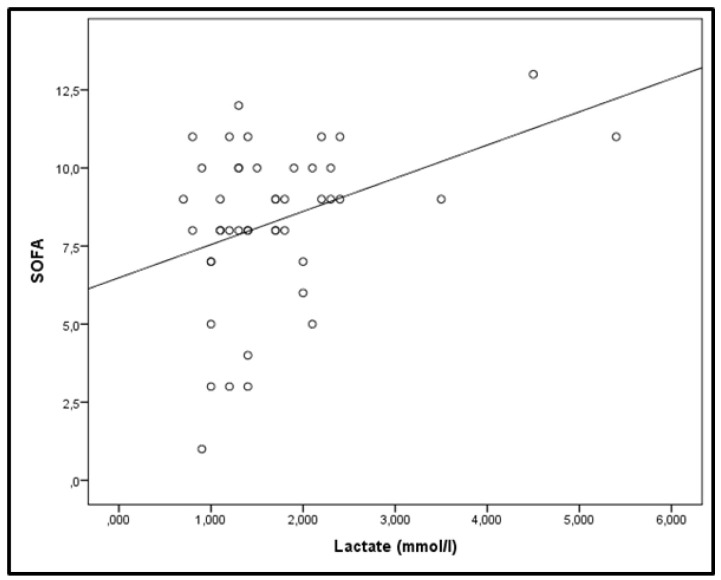
Correlation of lactate levels and SOFA scores on ICU admission. Correlations were performed by Spearman’s correlation coefficient. r^2^ = 0.33, *p* = 0.003.

Mixed model analysis revealed that the time variation of lactate levels was statistically significant (*p* = 0.001). The pattern of variation was significantly different among survivors and non-survivors (fixed-effects, Type III; *p* = 0.002). Increasing levels were observed in non-survivors, whereas lactate did not fluctuate during the 14-day study period in survivors (Figure 3a). Additionally, lactate levels were higher in non-survivors compared to survivors (the mixed-model showed mean lactate levels of survivors at 1.27 ± 0.05 mmol/L and of non-survivors, at 2.22 ± 0.12 mmol/L, *p* < 0.0001), and the differences were statistically significant on most days (Figure 3a). Moreover, as depicted in Figure 3b, hyperlactatemia (>2 mmol/L) occurred mostly in non-survivors. As seen, in 55% of the patients, lactate data were available beyond day 5. The mixed model was also applied to SOFA scores. SOFA scores measured on admission and thereafter on days 3, 5, 8, 10, 12, and 14 also varied with time (*p* < 0.001) and differed between survivors and non-survivors (Figure 4; *p* < 0.0001). The mixed-model showed SOFA scores of survivors at 6.6 and of non-survivors at 10.8. Additionally, the pattern of variation was significantly different (fixed-effects, Type III; *p* < 0.0001) with increasing SOFA scores in non-survivors and stable scores in survivors (Figure 4). Finally, correlation analysis performed between lactate levels and SOFA scores showed that, apart from day 14, lactate levels and SOFA scores correlated at all other time-points studied. More specifically, on admission: r^2^ = 0.33, *p* = 0.003 (Figure 5); day 3: r^2^ = 0.35, *p* = 0.002; day 5: r^2^ = 0.48, *p* = 0.003; day 8: r^2^ = 0.58, *p* < 0.0001; day 10: r^2^ = 0.51, *p* = 0.005, and day 12: r^2^ = 0.58, *p* = 0.009.

## 3. Discussion

The cornerstone of treatment in severely ill patients with COVID-19 infection is aggressive, supportive care, including mechanical ventilation and monitoring, since no efficacious treatment is available. The Surviving Sepsis Campaign COVID-19 panel recently issued guidelines on the management of critically ill adults with COVID-19 [15]. Mortality in COVID-19 ICU patients varies widely. A number of studies demonstrated mortality rates of 15–32% [6,16]. Others showed a mortality rate above 50% [17,18]. Extremely high mortality rates were found by Richardson et al. (88%) [5] and by Zhou et al. (78%) [19]. A systematic review summarized mortality rates and suggested that the overall ICU mortality is 25.7% and that poor outcomes in other studies were related to rationing of resources in overwhelmed ICUs [20]. Nevertheless, there is a need for further studies to draw firm conclusions on ICU mortality in patients with COVID-19.

In our small cohort consisting of 45 ICU patients, the vast majority of whom received mechanical ventilation (40/45, 89%), the 28-day mortality was 24.4% (11/45 patients died), indicating that many patients may survive critical illness. We are aware that with such a small sample size no definite conclusions on overall mortality rates can be drawn. In our country, to face the COVID-19 pandemic, ICU practitioners, hospital administrators and policy makers planned in advance for a substantial increase in critical care bed capacity. Our hospital had 30 ICU beds and 20 high-dependency beds before the pandemic. During the COVID-19 outbreak, the high-dependency unit was transformed into an ICU and another 24 beds were added to the existing ones—14 beds from the coronary unit and 10 beds from the recovery room. Thus, a total of 74 ICU beds were available to face the pandemic. Moreover, new equipment was provided, and physicians and nurses from other hospitals of the area of Athens were added to the existing staff.

A systematic review showed that the most reported predictors of poor prognosis in patients with COVID-19 are age, sex, C-reactive protein (CRP), lactate dehydrogenase (LDH), lymphocyte count, and features derived from CT scans [21]. COVID-19 patients treated in the ICU have a significantly higher level of inflammatory mediators than non-ICU patients, suggesting that a cytokine storm might be an underlying cause of disease severity [16]. Given the high mortality rates in these patients, early identification of prognostic indicators is crucial in guiding treatment and saving patients in a critical condition. A large study (*n* = 201) with COVID-19 ARDS-related pneumonia showed that high mortality was associated with older age, neutrophilia, and organ dysfunction [18]. A study on 217 ICU patients showed that mortality was significantly associated with older age, chronic renal disease, higher SOFA score, mechanical ventilation, vasopressors, and renal replacement therapy [22]. In our study, there was no association between comorbidities and clinical outcome. Another investigation reported that in COVID-19 ICU patients, APACHE II and SOFA scores were independent outcome predictors [23]. A retrospective, observational study of 85 fatal cases reported that the most common causes of death in COVID-19 ICU patients are respiratory failure (46.9%), septic shock (19.8%), multiple organ failure (16%), and cardiac arrest (8.6%). Acute coronary syndrome, malignant arrhythmia, and disseminated intravascular coagulation have been shown to be rare causes of death [24].

The possibility that high arterial blood lactate concentrations might be related to disease progression and outcome in COVID-19 ICU patients, as in other critical states, remains currently unclear. Tan and colleagues measured serial lactate levels from disease onset up to 40 days in 36 patients, out of whom 15 (42%) died. They found a narrow time window in which they could distinguish patients who died from those who were cured (*n* = 21). They concluded that lactic acid levels, in the presence of other variables, might be effective in predicting prognosis. Their findings, however, were rather descriptive and did not reach statistical significance [25].

In the present study, we measured lactate on admission in the ICU and thereafter every day, for a maximum of 14 days. Non-survivors had higher initial lactate levels than survivors. Cox regression analysis revealed that admission lactate levels constituted an independent outcome predictor in the presence of APACHE II. The AUC for lactate in predicting outcome was relatively high and similar to that of APACHE II, SOFA score, and troponin T. Based on the cut-off value of 1.85 mmol/L, lactate could predict patients’ death in our cohort of ICU COVID-19 patients. Moreover, the time course of blood lactate concentrations over the 14-day study period could distinguish survivors from non-survivors. Lactate increased in non-survivors, but remained essentially unchanged and within normal limits in survivors. Furthermore, all non-survivors had at some point an elevated lactate value during the course of their ICU stay. Of interest, the time course of the SOFA scores followed a similar pattern; non-survivors showed increased SOFA scores during the progression of the disease, whereas scores remained unchanged in survivors. These suggest that lactate kinetics mirror the degree of organ failure and are associated with poor outcomes in COVID-19 ICU patients. Sepsis-3 guidelines require blood lactate levels > 2 mmol/L to define septic shock, and sepsis is characterized by organ dysfunction, represented by an increase in the SOFA score of 2 points or more [13]. Blood lactate is easily obtained, and can be quickly measured at the patients’ bedside in the ICU, even prior to any invasive monitoring. The SOFA score can be rapidly calculated to describe organ dysfunction in the critically ill and its serial evaluation is recommended to monitor changes in patients’ status over time during an ICU stay [26].

In our study, of the 11 patients who were admitted in the ICU with high lactate, seven patients had septic shock and required vasopressors to maintain adequate arterial blood pressure. Elevated lactate in the ICU is a complex situation and may arise from an increase in lactate production, a decrease in lactate clearance due to renal or hepatic dysfunction, or their combination [7,8]. Although other processes may also increase lactate levels, i.e., increased aerobic glycolysis, beta-adrenergic stimulation, and the infusion of lactate-containing-intravenous solutions, the traditional view is that hyperlactatemia is attributed to anaerobic glycolysis due to inadequate perfusion and oxygen delivery to the tissues [7,8].

Our study has some limitations. First, this was a retrospective, observational, single-centre study, with a small sample size. Second, therapeutic interventions that might interfere with lactate levels, disease progression, and prognosis were not considered. Despite these, the present investigation is the first to raise the possibility that abnormal blood lactate kinetics are associated with adverse outcomes in COVID-19 ICU patients.

## 4. Materials and Methods

The study was approved by the Evangelismos Hospital Research Ethics Committee (170/24-4-2020) and all procedures carried out on patients were in compliance with the Helsinki Declaration. Informed written consent was obtained from all patients’ next-of-kin.

### 4.1. Study Population

This retrospective, observational study included all critically ill patients suffering from COVID-19 pneumonia hospitalized in the ICU of the Evangelismos General Hospital from 14 March to 9 May 2020. SARS-CoV-2 infection was diagnosed by real-time reverse transcription PCR (RT-PCR) in nasopharyngeal swabs. Following study enrolment, demographic characteristics, comorbidities, symptoms, vital signs, laboratory findings, and COVID-19-targeted compounds were recorded. Acute physiology and chronic health evaluation (APACHE II) was calculated on admission into the ICU. Sequential organ failure assessment (SOFA) score was assessed on admission and on days 3, 5, 8, 10, 12, and 14. Acute respiratory distress syndrome (ARDS) was assessed according to the Berlin definition [27]. Sepsis and septic shock were defined according to Sepsis-3 guidelines [13]. Outcome was defined as 28-day ICU mortality.

### 4.2. Lactate Measurement

Blood lactate was measured on admission into the ICU and thereafter daily up to day 14. Two to three measurements per day were available and the highest or mean daily value was taken into account for statistical analyses. Lactate measurements of days 1 and 2 were combined due to the fact that admission to the ICU occurred mostly in late afternoon. A blood gas analyzer (Radiometer ABL 700 series, Radiometer Medical APS, Copenhagen, Denmark) was used for lactate measurements.

### 4.3. Statistical Analysis

Data are presented as mean ± standard deviation (SD) for normally distributed variables or as median with inter-quartile range (Q1–Q3) for skewed data. The two-group comparisons were performed by the t-test or the non-parametric Mann-Whitney test. Associations between qualitative variables were examined by the chi-squared test or the Fisher exact when appropriate. Correlations were performed by Spearman’s correlation coefficient. Univariate Cox regression models were fitted for statistically significant variables by the two-group comparisons, except from those that are included in the APACHE II score, to examine their relationship with time to 28-day ICU mortality. A multivariate Cox regression model was subsequently performed with covariates which were not correlated. Results are described by the relative risk (RR) and 95% confidence interval (CI). Receiver operating characteristic (ROC) curves were plotted thereafter using 28-day ICU mortality as the classification variable. The optimal cut-off value for predicting 28-day mortality was calculated as the point with the greatest combined sensitivity and specificity. Comparison of the ROC curve areas were performed with the z-statistic test of Hanley and McNeil. The Kaplan-Meier method was used for 28-day ICU mortality absence probability estimation and the log-rank test for comparison of the two groups. Mixed error-component models were used to describe the time progression of variables, namely lactate and SOFA score. Since some patients died or were extubated at different time-points, measurements were not available for all time-points in all subjects and thus a general linear model (GLM) could not be applied to analyze repeated lactate measurements. Instead, we used the mixed model analysis. We defined 28-day ICU mortality as the dependent variable and both the mean lactate values (or SOFA, accordingly) and time-points as fixed variables, applying a factorial model. We chose as the most appropriate covariance structure the one with the smaller Schwarz Bayesian information criterion (BIC), that is the first-order autoregressive variance-covariance structure. models included 28-day ICU outcome (survivors/non-survivors), time and their interaction (mixed model fixed effects analysis). The analyses were performed with the IBM SPSS statistical package, version 22.0 (IBM Software Group, Armonk, NY, USA), and GraphPad Prism, version 8.0 (GraphPad Software, San Diego, CA, USA). All *p*-values are two-sided; *p* < 0.05 was considered significant.

## 5. Conclusions

The data presented support the usefulness of initial and repeated lactate measurements in critically ill patients with COVID-19 pneumonia requiring intensive care. Admission lactate constituted an independent predictor for 28-day mortality in the presence of APACHE II. The time course of lactate mirrors the degree of organ dysfunction, as assessed by serial determinations of the SOFA score, and distinguishes survivors from non-survivors.

## Figures and Tables

**Table 1:** Clinical characteristics and laboratory data in critically ill patients on admission to the ICU. Patients’ outcome and demographics are also shown.

Characteristics	All Patients	Survivors	Non-Survivors	*p*-Value
Number of patients, N (%)	45	34 (75.6%)	11 (24.4%)	
Age (years), (mean ± SD)	64 ± 10	63 ± 10	67 ± 10	0.244
Sex, N (%)				0.765
Male	33 (73.3%)	24 (72.7%)	9 (27.3%)	
Female	12 (26.7%)	10 (83.3%)	2 (16.7%)	
Comorbidities, N (%)				0.887
Hypertension	18	15 (83.3%)	3 (16.7%)	
Diabetes	5	3 (60.0%)	2 (40.0%)	
Coronary artery disease	3	1 (33.3%)	2 (66.7%)	
COPD	1	0 (0.0%)	1 (100.0%)	
Asthma	1	1 (100.0%)	0 (0.0%)	
Hyperlipidaemia	10	8 (80.0%)	2 (20.0%)	
Chronic kidney disease	1	0 (0.0%)	1 (100.0%)	
Hepatitis	1	1 (100.0%)	0 (0.0%)	
Characteristics on ICU Admission				
APACHE II score, (median, IQR)	17 (14–19)	16 (14–18)	18 (16–22)	0.025 *
SOFA score, (median, IQR)	9 (8–10)	8 (7–10)	10 (8–11)	0.031 *
PaO_2_/FiO_2_ (mmHg), (mean ± SD)	166 ± 70	171 ± 71	151 ± 70	0.418
PCO_2_ (mmHg), (mean ± SD)	45 ± 9	44 ± 7	50 ± 13	0.051
pH, (median, IQR)	7.33 (7.29–7.45)	7.36 (7.31–7.45)	7.31 (7.25–7.38)	0.063
HCO_3_ (mEq/L), (median, IQR)	23 (21–27)	23 (21–25)	21 (21–30)	0.474
Vitals signs				
Heart rate (bpm), (mean ± SD)	84 ± 28	87 ± 25	77 ± 37	0.247
Mean Arterial pressure (mmHg), (median, IQR)	78 (70–90)	77 (70–84)	78 (68–105)	0.71
Respiratory rate (breaths/min), (mean ± SD)	22 ± 3	21 ± 3	24 ± 3	0.023 *
Temperature (°C), (mean ± SD)	37.0 ± 1.4	37.3 ± 1.2	36.0 ± 1.6	0.0051 *
Laboratory data				
Haemoglobin, (mean ± SD)	12.4 ± 2.2	12.8 ± 1.8	11.2 ± 2.8	0.033 *
Haematocrit, (mean ± SD)	37.3 ± 6.3	38.6 ± 4.7	33.4 ± 8.8	0.015 *
White Blood Cell count (per μL), (median, IQR)	9400 (5200–11,750)	9050 (5275–10,868)	12,800 (1300–154,100)	0.572
Neutrophils (%), (median, IQR)	82.0 (78.0–87.5)	81.5 (78.6–87.0)	86.0 (72.0–90.0)	0.891
Lymphocytes (%), (median, IQR)	13.0 (7.5–18.5)	13.0 (8.0–18.3)	9.0 (5.0–21.0)	0.561
Platelets (per μL), (mean ± SD)	196,844 ± 85,148	207,912 ± 63,197	162636 ± 130449	0.127
PT (sec), (median, IQR)	13 (13–14)	13 (13–14)	13 (12–14)	0.923
APTT (sec), (mean ± SD)	34.3 ± 5.5	34.3 ± 5.8	34.5 ± 5.1	0.923
INR, (median, IQR)	1.06 (1.00–1.10)	1.06 (1.00–1.10)	1.07 (1.00–1.13)	0.881
Creatinine (mg/dL), (mean ± SD)	1.1 ± 0.5	0.9 ± 0.2	1.5 ± 0.8	0.0003 *
Glucose (mg/dL), (median, IQR)	142 (117–189)	142 (116–186)	145 (117–262)	0.525
Total Bilirubin (mg/dL), (median, IQR)	0.6 (0.5–0.8)	0.6 (0.5–0.8)	0.5 (0.4–0.8)	0.321
Albumin (g/dL), (mean ± SD)	3.4 ± 0.5	3.4 ± 0.5	3.1 ± 0.5	0.129
Globulin (g/dL), (mean ± SD)	2.6 ± 0.5	2.7 ± 0.5	2.3 ± 0.7	0.064
CKMB (IU/L), (median, IQR)	24.0 (15.5–32.5)	23.5 (14.8–33.0)	25.0 (20.0–32.0)	0.711
CK (U/L), (median, IQR)	159.0 (55.5–365.0)	169.5 (63.8–364.8)	137.0 (21.0–376.0)	0.493
Fibrinogen (mg/dL), (mean ± SD)	632.6 ± 166.3	652.7 ± 157.7	570.5 ± 184.5	0.157
CRP (mg/dL), (mean ± SD)	17.0 ± 11.6	15.9 ± 9.3	20.4 ± 17.1	0.269
γ-GT (IU/L), (median, IQR)	59.0 (27.0–113.0)	62.0 (27–122.5)	42.0 (24.0–78.0)	0.225
Urea (mg/dL), (median, IQR)	34.0 (27.0–49.5)	31.0 (24.8–41.0)	46.0 (39.0–89.0)	0.0013 *
AST (IU/L), (median, IQR)	42.0 (28.5–58.5)	42.5 (33.8–60.0)	28.0 (26.0–58.0)	0.274
ALT (IU/L), (mean ± SD)	39.2 ± 20.9	40.4 ± 20.4	35.6 ± 23.1	0.506
Na^+^ (mEq/L), (mean ± SD)	137.9 ± 5.8	138.0 ± 5.1	137.5 ± 8.0	0.779
K^+^ (mg/dL), (mean ± SD)	4.1 ± 0.7	4.2 ± 0.7	3.8 ± 0.7	0.117
ALP (U/L), (median, IQR)	67.0 (45.0–105.0)	65.5 (43.8–130.8)	67.0 (52.0–102.0)	0.740
LDH (U/L), (median, IQR)	479.0 (347.0–631.5)	482.5 (356.5–630.3)	470.0 (235.0–637.0)	0.636
High-sensitive troponin T (ng/mL), (median, IQR)	15 (10–42)	12 (10–28)	33 (16–83)	0.013 *
Amylase (U/L), (median, IQR)	65.0 (38.5–97.8)	66.0 (38.0–106.0)	56.0 (40.0–97.0)	0.693
Lactate (mmol/L)	1.4 (1.1–2.1)	1.4 (1.0–1.8)	2.0 (1.4–2.3)	0.0066 *
COVID-19-Targeted Treatment				0.81
Azithromycin/chloroquine/lopinavir/ritonavir	20	17	3	
Azithromycin/chloroquine	15	10	5	
Lopinavir/ritonavir/chloroquine	5	4	1	
Chloroquine	4	2	2	
Plasma	1	1	0	
Outcomes				
LoS in the ICU (days), (median, IQR)	13.0 (8.0–17.0)	13.0 (10.0–19.0)	8.0 (4.0–11.0)	0.007 *
Mechanical ventilation, N (%)	39	28	11	ns
Duration of mechanical ventilation (days), (median, IQR)	10.0 (4.0–14.0)	10.0 (3.0–16.0)	8.0 (4.0–11.0)	ns

* *p*-value < 0.05. Data are expressed as number of patients (N), percentages of total related variable (%) and mean ± SD for normally distributed variables and median (IQR) for skewed data. Patients were divided into 2 groups, depending on 28-day ICU mortality. For differences between the 2 groups, either the Student’s t-test for normally distributed data or the Mann–Whitney test for skewed data was used. Vital signs listed are the most abnormal recorded during the 24-h post-admission. Laboratory data were measured once (within 24-h from admission), apart from lactate, which was measured two to three times daily and the highest admission value is listed (note that lactate measurements of days 1 and 2 were combined due to the fact that admission to the ICU occurred mostly in late afternoon). Definition of abbreviations: γ-GT = γ-Glutamyl transpeptidase; ALP = alkaline phosphatase; ALT = alanine transaminase; APACHE = acute physiology and chronic health evaluation; APTT = activated partial thromboplastin time; AST= aspartate transaminase; CAD = coronary artery disease; CK = creatine kinase; CKD = chronic kidney failure; CKMB = creatine kinase myocardial band; COPD = chronic obstructive pulmonary disease; CRP = C-reactive protein; ICU = intensive care unit; INR = international normalized ratio; LDH = lactate dehydrogenase; LoS = length of stay; PT = prothrombin time; SOFA = sequential organ failure assessment.

**Table 2:** Univariate Cox regression analysis for 28-day ICU mortality in COVID-19 patients.

Variable	Univariate RR	95% CI	*p*
APACHE II score (per 1 unit increase)	1.235	1.033–1.476	0.020 *
SOFA score (per 1 unit increase)	1.424	0.939–2.160	0.096
Lactate (per 0.1 mmol/L increase)	2.911	1.552–5.460	0.001 *
Troponin T (per 1 ng/mL)	1.004	1.001–1.007	0.015 *

* *p*-value < 0.05. Univariate Cox regression analysis-derived relative risk (RR) and 95% confidence intervals (95% CI). Cox regression models were fitted for the four variables to examine their relationship with time to 28-day ICU mortality. All measurements were taken up to 24-h after ICU admission. Lactate measurements of days 1 and 2 were combined because admission to the ICU occurred mostly in late afternoon. APACHE = acute physiology and chronic health evaluation; CI = confidence interval; ICU = intensive care unit; RR = relative risk; SOFA = sequential organ failure assessment.

## Supplementary Materials

The following are available online at https://www.mdpi.com/2218-1989/10/10/386/s1.
